# Neural basis of induced phantom limb pain relief

**DOI:** 10.1002/ana.25371

**Published:** 2019-01-07

**Authors:** Sanne Kikkert, Melvin Mezue, Jacinta O'Shea, David Henderson Slater, Heidi Johansen‐Berg, Irene Tracey, Tamar R. Makin

**Affiliations:** ^1^ Wellcome Centre for Integrative Neuroimaging, FMRIB Centre, Nuffield Department of Clinical Neurosciences University of Oxford Oxford United Kingdom; ^2^ Donders Institute for Brain, Cognition and Behaviour Radboud University Nijmegen Nijmegen the Netherlands; ^3^ Neural Control of Movement Laboratory, Department of Health Sciences and Technology ETH Zürich Zürich Switzerland; ^4^ Oxford Centre for Enablement, Nuffield Orthopaedic Centre Oxford United Kingdom; ^5^ Institute of Cognitive Neuroscience University College London London United Kingdom; ^6^ Wellcome Centre for Human Neuroimaging University College London London United Kingdom

## Abstract

**Objective:**

Phantom limb pain (PLP) is notoriously difficult to treat, partly due to an incomplete understanding of PLP‐related disease mechanisms. Noninvasive brain stimulation (NIBS) is used to modulate plasticity in various neuropathological diseases, including chronic pain. Although NIBS can alleviate neuropathic pain (including PLP), both disease and treatment mechanisms remain tenuous. Insight into the mechanisms underlying both PLP and NIBS‐induced PLP relief is needed for future implementation of such treatment and generalization to related conditions.

**Methods:**

We used a within‐participants, double‐blind, and sham‐controlled design to alleviate PLP via task‐concurrent NIBS over the primary sensorimotor missing hand cortex (S1/M1). To specifically influence missing hand signal processing, amputees performed phantom hand movements during anodal transcranial direct current stimulation. Brain activity was monitored using neuroimaging during and after NIBS. PLP ratings were obtained throughout the week after stimulation.

**Results:**

A single session of intervention NIBS significantly relieved PLP, with effects lasting at least 1 week. PLP relief associated with reduced activity in the S1/M1 missing hand cortex after stimulation. Critically, PLP relief and reduced S1/M1 activity correlated with preceding activity changes during stimulation in the mid‐ and posterior insula and secondary somatosensory cortex (S2).

**Interpretation:**

The observed correlation between PLP relief and decreased S1/M1 activity confirms our previous findings linking PLP with increased S1/M1 activity. Our results further highlight the driving role of the mid‐ and posterior insula, as well as S2, in modulating PLP. Lastly, our novel PLP intervention using task‐concurrent NIBS opens new avenues for developing treatment for PLP and related pain conditions. **ANN NEUROL 2019;85:59–73.**

Neuropathic pain following peripheral nerve injury is typically unresponsive to conventional analgesic treatments and poses a significant medical problem.^1^ Phantom limb pain (PLP) following amputation is an archetypal example of an intractable neuropathic pain syndrome. An improved understanding of the neural circuits underlying neuropathic pain could facilitate targeted treatments for such conditions.^2^

Several innovative PLP treatments have been designed based on putative PLP mechanisms (eg, brain stimulation, mirror therapy, virtual reality, graded motor imagery).^3^, ^4^, ^5^, ^6^ These innovative treatments inspired development of nonconventional therapies for other neuropathic pain conditions (eg, complex regional pain syndrome, chronic back pain).^6^, ^7^ However, despite some evidence of efficacy, although limited, there remains no consensus on PLP management.^8^, ^9^, ^10^ Furthermore, the methodological quality and strength of this previous evidence is variable, with many studies omitting the use of blinding, control for placebo effects, and validation against control interventions. Importantly, a major challenge in developing targeted PLP treatments stems from an incomplete understanding of its neural basis.

Noninvasive brain stimulation (NIBS) techniques such as transcranial direct current stimulation (tDCS) have previously been used to reduce pain in various chronic intractable pain syndromes,^11^ including PLP,^5^, ^12^ although it remains unclear how NIBS induces pain relief.^5^, ^12^, ^13^, ^14^ tDCS is thought to cause widespread cortical and subcortical effects that propagate beyond the designated stimulation area.^15^, ^16^, ^17^ tDCS effects may be state‐dependent; for example, combining stimulation with a meaningful task can enhance or prolong the effects of tDCS on learning and memory.^18^, ^19^, ^20^ It has been proposed that by activating task‐dependent functional networks, concurrent NIBS can facilitate the processes most relevant for these activated networks. As such, task‐concurrent NIBS offers potential avenues for targeting functional networks more specifically.^21^ However, recent criticism has been mounting to challenge the mechanistic validity of tDCS.^22^, ^23^, ^24^ To achieve treatment that is both effective and generalizable to related syndromes, it is essential to consider both disease and treatment mechanisms.

In the past decades, there has been an almost exclusive focus on maladaptive plasticity in the primary somatosensory (S1) and motor cortex (M1) as a key PLP mechanism^25^, ^26^, ^27^, ^28^, ^29^, ^30^: Following arm amputation, the missing hand representation degrades and the neighboring facial representation invades the freed‐up cortical territory. However, we have previously been unable to find lip or residual‐limb remapping into the M1/S1 missing hand cortex, or a relationship between lip/residual‐limb representation and PLP.^27^, ^28^, ^29^ Conversely, recent evidence of stronger maintained phantom hand representation in amputees with worse chronic PLP led us to propose an alternative PLP mechanism of maintained representation.^27^, ^29^, ^31^, ^32^ Maintained activity in the S1/M1 missing hand area (suggested to be driven by ectopic peripheral inputs^29^, ^31^ or prediction errors resulting from a phantom hand perception in the absence of a physical stimulus^33^) may be aberrant, leading to broad‐scale functional reorganization at a network level.^34^ This alternative mechanism offers new potential targets for PLP treatment, as it may be possible to exploit it to alter large‐scale cortical plasticity.

Here, we investigated whether targeting phantom hand representation produced PLP relief using a within‐participants, double‐blind, and sham‐controlled design. To influence information processing of peripheral missing hand signals (previously associated with PLP), we instructed amputees to execute phantom hand movements during tDCS.^27^, ^29^ We applied excitatory tDCS over the S1/M1 missing hand cortex, as previously implemented for related neuropathic pain conditions.^5^, ^11^, ^12^ Neuroimaging was used during and after tDCS to evaluate the neural underpinnings of PLP relief.

## Subjects and Methods

Full details of the experimental protocol, data, and supplementary analysis and results are available on https://osf.io/4a5zg/. Here, we focus on the procedures relevant to the key analyses described below.

### *Participants and Study Design*

Seventeen unilateral upper‐limb amputees experiencing PLP on a weekly basis (mean age ± standard error of the mean = 47 ± 3 years, 6 right‐arm amputees, 4 females; Table 1) and 15 age‐matched controls (2‐handers, age = 46 ± 3 years, 7 dominant left‐handers, 4 females) were recruited. Six amputees participating in this study also participated in our previous study 3 years earlier,^27^ although note that novel data were acquired for all participants. Exclusion criteria included magnetic resonance imaging (MRI) or tDCS contraindications, intake of γ‐aminobutyric acid (GABA)–affecting medications, and age (< 18 or > 70 years). Ethical approval was granted by the National Health Service (NHS) Research Ethics service (Ref: 10/H0707/29), and written informed consent was obtained from all participants prior to study onset. Two amputees were excluded from the study prior to completion, due to safety considerations and an inability to perform the phantom hand movement task (these data are therefore not reported in the current article).

**Table 1:** Demographic and Clinical Details

Patient	Age, yr	Age at Amp., yr	Amp. Level	Side/Dominant	Chronic PLS	Chronic PLP	Average PLP	Chronic Residual Arm Pain	Cause of Amp.	Pros. Usage
A01	43	26	2	R/R	90	70	50	0	Trauma	5
A02	68	53	2	R/R	25	42.5	50	0	Trauma	5
A03	36	31	2	R/L	20	40	30	80	Trauma	0
A04	54	54	2	L/R	90	10	40	20	Vascular	3
A05	28	24	1	L/R	15	26.7	20	5	Trauma	3
A06	52	28	4	L/R	80	35	30	10	Trauma	5
A07	49	45	2	L/L	80	70	50	10	Tumor	3
A08	47	17	2	L/R	100	15	20	3.3	Trauma	2
A09	48	27	2	R/R	100	45	70	0	Trauma	0
A10	23	18	4	R/R	90	25	25	0	Trauma	0
A11	49	19	2	L/R	70	50	40	0	Trauma	5
A12	60	31	2	L/R	70	12.5	20	0	Trauma	0
A13	56	20	5	L/L	70	70	30	0	Trauma	5
A14	40	27	2	R/L	100	80	20	26.7	Trauma	2
A15	45	38	4	L/R	89	92	40	17.5	Trauma	2

Yr. = year; Amp. = amputation; Amp. Level: 1 = shoulder, 2 = above elbow, 3 = through elbow, 4 = below elbow, 5 = wrist and below; Dominant = hand dominance prior to amputation (based on self‐report); L = left, R = right; PLP = phantom limb pain; PLS = phantom limb sensation; Pros. Usage = prosthetics usage: 0 = never, 1 = rarely, 2 = occasionally, 3 = daily (<4 hours), 4 = daily (>4 hours), 5 = daily (>8 hours); Side = side of amputation; Vascular = vascular disease.

Amputees participated in 4 consecutive experimental sessions, spaced at least 1 week apart. One amputee completed only 3 experimental sessions (see Supplementary Table 1 for number of participants per assessment). Here, we detail methods and results related to our intervention and sham condition. We included 2 further active tDCS control conditions in this study, the results of which are described in Supplementary Tables 2 and 3: (1) anodal tDCS over S1/M1 contralateral to the intact hand, designed to control for stimulation site; and (2) cathodal tDCS over the S1/M1 missing hand cortex, designed to control for current direction. Participants were informed in advance that they would undergo different types of brain stimulation that may either be active or not, without specifying the distribution of active/control treatments. Control participants were tested once without NIBS to enable baseline comparisons with the amputees. Amputees’ phantom hand was matched to controls’ nondominant hand (note that similar results are found when matching amputees’ phantom hand to controls’ dominant hand; see https://osf.io/4a5zg/).

The 4 conditions were counterbalanced across participants. Chi‐squared testing confirmed that the stimulation conditions were preceded equally often by the other conditions (intervention: ${\text{χ}}_{\left[3\right]}^{2}$ = 0.20, *p* = 0.978; sham: ${\text{χ}}_{\left[3\right]}^{2}$ = 0.20, *p* = 0.978; control site: ${\text{χ}}_{\left[3\right]}^{2}$ = 0.29, *p* = 0.963; cathodal: ${\text{χ}}_{\left[3\right]}^{2}$ = 0.73, *p* = 0.865).

### *Transcranial Direct Current Stimulation*

A DC‐Stimulator (Magstim, Whitland, UK) was used to deliver electric current to the brain via two 5 × 7cm electrodes (Easycap, Wörthsee, Germany), fitted with 5kΩ MRI‐compatible resistors. High‐chloride electrolyte electroencephalographic (EEG) gel was used as a conducting medium between the scalp and the electrodes. Electrode placement followed standard procedures and was determined according to the EEG 10‐20 system.^35^ For intervention stimulation, the anodal electrode was placed over the S1/M1 missing hand cortex (5cm lateral to Cz, corresponding to C3/C4), with the cathodal electrode placed over the contralateral supraorbital area (and vice versa for cathodal control stimulation). Stimulation lasted 20 minutes (fade‐in/fade‐out phases = 10 seconds), with 1mA intensity. For sham (and control site) stimulation, the electrodes were positioned over the intact hand S1/M1 and the contralateral supraorbital area, but in the sham condition the stimulator was turned off after impedance was stabilized (after ∼30 seconds). This standard procedure ensures that any sensation experienced during impedance stabilization is similar across conditions, maximizing successful blinding. To prevent the participant's or experimenter's awareness of reddening of the skin underneath the electrode, which can be greater in active tDCS conditions,^36^ we placed a large swimmer's cap over the participant's head immediately after electrode positioning, which was kept on until ∼90 minutes after stimulation offset.

### *Pain Ratings*

At study onset, amputees rated PLP frequency experienced within the past year and worst PLP intensity experienced during a typical week. Chronic PLP was calculated by dividing worst PLP intensity (scale 0–100, ranging from no pain to worst pain imaginable) by PLP frequency (1, all the time; 2, daily; 3, weekly; 4, several times per month; and 5, once or less per month). Note that we have previously shown excellent interstudy consistency for this measure of PLP chronicity.^37^

**Figure 1:**
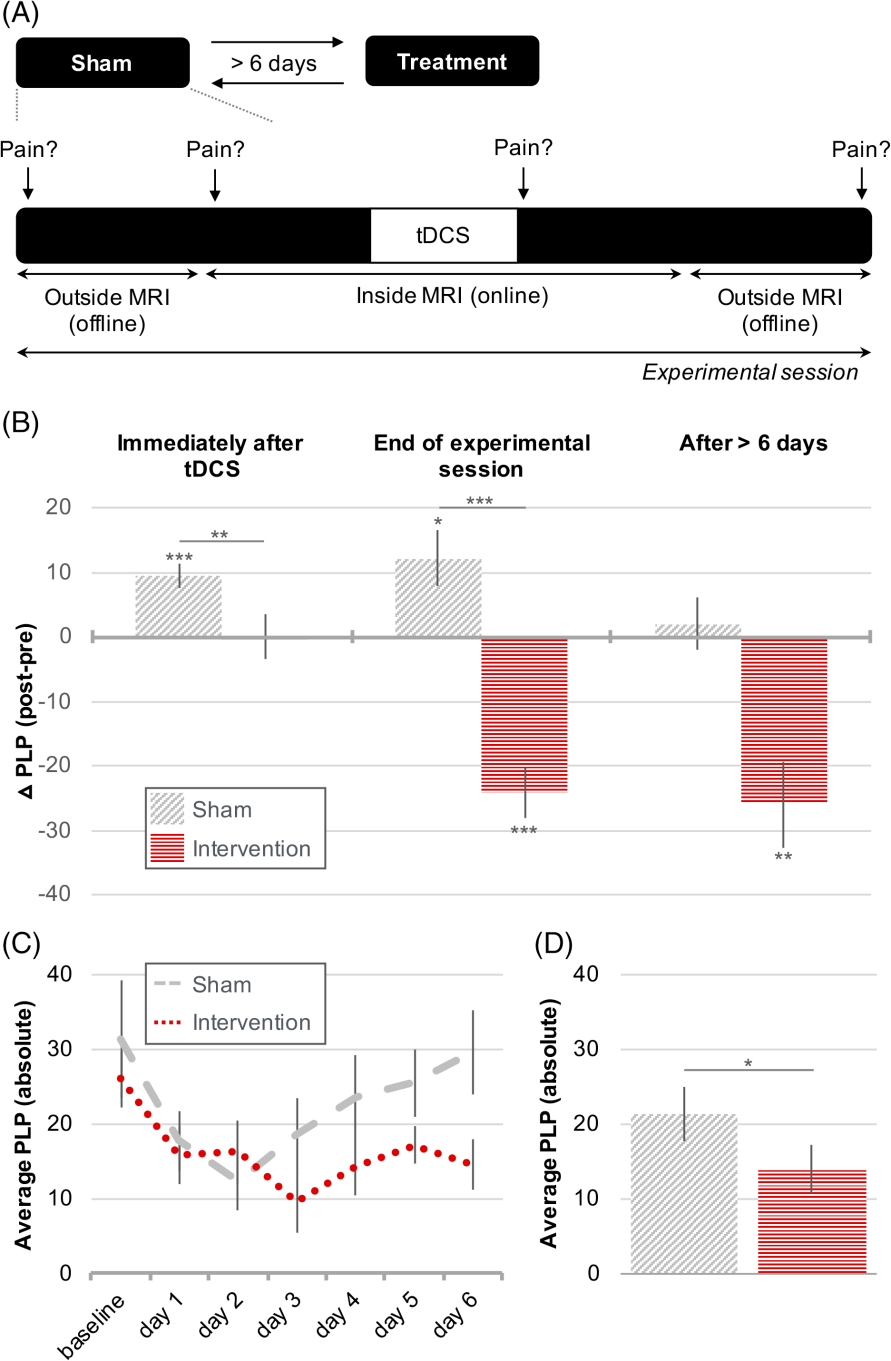
Noninvasive brain stimulation relieves phantom limb pain (PLP) with effects lasting up to 1 week. (A) Experimental timeline. Transient PLP ratings were obtained at 4 different time points within each experimental session. To account for interparticipant variability, post‐stimulation online and offline PLP ratings were contrasted with their respective baselines. To examine longer‐term effects of stimulation, participants were also asked to respond to daily text messages requesting PLP ratings for 6 days post stimulation. (B) Task‐concurrent intervention stimulation effects on PLP, assessed in the experimental sessions. Positive values on the y‐axis indicate PLP increases, and negative values indicate PLP relief. The sham condition is depicted using diagonal shading and the intervention condition using horizontal shading. (B, left) Whereas PLP was increased in the sham condition immediately after stimulation, no PLP increase was observed in the intervention condition, leading to a significant difference between conditions. (B, middle) At the end of the experimental session, PLP remained increased in the sham condition, whereas significant PLP relief was observed after intervention stimulation. The PLP change was significantly different between conditions. (B, right) At least 1 week after stimulation, there was no longer a change in PLP in the sham condition, but significant PLP relief was still observed for the intervention condition. (C, D) A second measure of week‐long noninvasive brain stimulation effects on PLP was obtained using text messages, asking amputees to rate their average PLP on a daily basis. Note that no difference scores were calculated for this measure, and as such, the y‐axis reflects absolute PLP ratings. (C) PLP ratings over the course of 6 days post stimulation. Dashed line = sham stimulation; dotted line = intervention stimulation. (D) The results in C tested as a main effect. In the week after stimulation, PLP was significantly lower in the intervention condition compared to sham. Together, these results show that a single 20‐minute application of task‐concurrent intervention stimulation relieved PLP, with effects lasting up to 1 week post stimulation. Asterisks indicate statistical significance: *corrected *p* < 0.05, **corrected *p* < 0.01, ***corrected *p* < 0.001. Error bars represent the standard error of the mean. MRI = magnetic resonance imaging; tDCS = transcranial direct current stimulation. [Color figure can be viewed at www.annalsofneurology.org]

Ratings of transient PLP intensity (scale 0–100, as above) were obtained using a short pain questionnaire (SPQ), administered throughout each experimental session (Fig 1A). To assess PLP outside the MRI scanner (“offline”), participants completed the SPQ in writing. Inside the MRI scanner (“online”), an experimenter blinded to the tDCS condition verbally administered the SPQ. Additionally, participants were sent automated daily text messages, requesting an average PLP intensity rating. These texts started 1 week before experimental onset, and ended 1 week after the final experimental session.

No differences were found in baseline PLP levels across the 4 stimulation conditions (online: ${\text{χ}}_{\left[3\right]}^{2}$ = 5.81, *p* = 0.12; offline: ${\text{χ}}_{\left[3\right]}^{2}$ = 3.13, *p* = 0.37). To examine stimulation effects, baseline PLP levels were subtracted from post‐stimulation PLP ratings. Online PLP measures assess PLP effects immediately after stimulation offset (ie, *PLP ∼0 minutes after stimulation offset − PLP ∼20 minutes prior to stimulation onset*). Offline PLP measures assess PLP effects at the end of the experimental session (ie, *PLP ∼90 minutes after stimulation offset − PLP ∼90 minutes prior to stimulation onset*). Lastly, to examine PLP effects lasting 1 week post stimulation, we took 2 approaches: (1) we contrasted the first baseline (offline) PLP rating obtained in the following experimental session with the pre‐stimulation (offline) PLP rating (ie, *PLP > 6 days after stimulation offset − PLP ∼90 minutes prior to stimulation onset*); and (2) we used the daily text messages data as an absolute PLP measure (no difference scores were created for this data).

A linear regression analysis was conducted on the resulting PLP scores to remove the influence of chronic PLP from the transient PLP ratings. This ensured that the intervention effects were not masked by the high interparticipant variability in chronic PLP and better characterized fluctuations in transient PLP. We conducted all statistical analysis (both stimulation‐induced PLP effects and correlations with functional MRI measures) using the residuals of this regression analysis.

### *Scanning Procedures*

#### Body Localizer Scans

Body localizer scans were obtained at baseline in the first experimental session and post stimulation in each experimental session. During the body localizer scan, participants were visually instructed (at 0.5Hz pace) to move their intact hand (all fingers flexion/extension), phantom hand (as the intact hand), feet (bilateral toes), and lips in a blocked design. Each movement condition was repeated 4 times in a semicounterbalanced protocol, alternating 12 seconds of movement and rest.

Importantly, phantom hand movements are distinguishable from imagined movements, in terms of both central and peripheral motor signals.^37^, ^38^, ^39^, ^40^ Participants were therefore clearly instructed to make actual instead of imagined phantom hand movements, and to attempt performing the instructed movements if phantom movements were restricted. Instructions were delivered visually using Presentation software (NeuroBehavioral systems, Berkeley, US; v16.4) via a mirror mounted on the head coil.

#### Resting State Scans

For the resting state scan, participants were instructed to lie still, stay awake, keep their eyes open, stare at a fixation cross on the screen, and let their mind wander. This scan was obtained at baseline in the first experimental session.

#### Stimulation Scans

During stimulation, participants were visually instructed to perform phantom hand movements in a block design, alternating 45 seconds of movement with 15 seconds of rest. The instructed movements were wrist flexing, all fingers flexion/extension, index finger flexion/extension, ring finger flexion/extension, and fingers adduction. Participants were instructed to make as many movements as possible in each 45‐second block, at a comfortable pace. Half of the blocks involved phantom hand movements only. In the other half, participants used their intact hand to mirror the precise degree and speed of the phantom hand movement. Movements made during stimulation were video‐recorded and inspected offline by a blinded experimenter for a subset of 11 amputees. In this video analysis, we focused on the mirrored phantom hand movements during hand opening/closing and ring finger flexion movements, representing a relatively easy and more difficult phantom hand movement (respectively). Hand movements were rated from full (1) to little or no visible movement (4; see https://osf.io/4a5zg/). Importantly, no significant differences in movement scores were found between the stimulation conditions (phantom hand opening/closing: ${\text{χ}}_{\left[3\right]}^{2}$ = 2.68, *p* = 0.443; phantom ring finger flexion: ${\text{χ}}_{\left[3\right]}^{2}$ = 1.04, *p* = 0.792), demonstrating that any differences between stimulation sessions were not caused by differences in task performance.

### *MRI Data Acquisition*

MRI images were collected using a 3T Verio MRI scanner (Siemens, Erlangen, Germany) and a 32‐channel head‐coil. A T1‐weighted sequence was used to acquire structural images (repetition time [TR] = 2,040 milliseconds, echo time [TE] = 4.7 milliseconds, flip angle = 8°, voxel size = 1mm^3^). The body localizer and resting state scans were obtained using a multiband T2*‐weighted pulse sequence with an acceleration factor of 6 (TR = 1,300 milliseconds, TE = 40 milliseconds, voxel size = 2mm^3^, flip angle = 66°, 72 transversal slices, 314/230 volumes respectively). A high‐saturation first volume was collected for each multiband run for registration purposes. During stimulation, an echo‐planar T2*‐weighted pulse sequence was used (TR = 2,000 milliseconds, TE = 30 milliseconds, flip angle = 90°, voxel size = 3mm^3^, 192 transversal slices, 608 volumes).

### *MRI Analysis*

All imaging data were processed using FSL v5.0 (https://fsl.fmrib.ox.ac.uk/fsl/fslwiki).^41^ Data collected for individuals with an amputated right hand (or dominant left hand for controls) was flipped on the midsagittal plane before all analyses to align the phantom hand hemisphere. To ensure that this procedure did not impact our findings, a regressor of no interest for brain flipping was added to our main analysis (note that adding this regressor did not impact our findings; see https://osf.io/4a5zg/ for analogous results without this “flipping” regressor). To investigate neural activity in brain regions without strong lateralization, the nonflipped data were also analyzed.

#### Body Localizer Scans

Common preprocessing steps were applied using FSL's Expert Analysis Tool FEAT (v6.00): motion correction using Functional Magnetic Resonance Imaging of the Brain's Linear Image Registration Tool (MCFLIRT), brain extraction using the automated brain extraction tool BET, spatial smoothing using a 3mm full width at half maximum (FWHM) Gaussian kernel, and high‐pass temporal filtering with a cutoff of 100 seconds.

Functional images were aligned to the structural image, initially using linear registration (FLIRT) and optimized using boundary‐based registration. Functional images acquired post stimulation were first registered to the example functional image acquired in the baseline experimental session (7 degrees of freedom). Structural images were transformed to Montreal Neurological Institute (MNI) standard space using nonlinear registration (FNIRT), and the resulting warp fields were applied to the functional statistical images. Time series statistical analysis was done using FILM with local autocorrelation correction. First‐level (time series) parameter estimates were computed using a voxel‐based general linear model based on the double‐gamma hemodynamic response function, its temporal derivatives, and estimated head motion parameters (MCFLIRT). Data were further assessed for excessive motion, and volumes with an estimated absolute mean displacement > 1mm (half of the functional voxel size) were scrubbed (maximum percentage of volumes scrubbed in a scan = 1% and 2% for baseline and post stimulation sessions, respectively).

Group‐level analysis was performed using FMRIB's Local Analysis of Mixed Effects (FLAME). An averaged whole‐brain map was created for the amputees’ phantom (or nondominant) hand versus feet movement contrast. A regressor of no interest for brain flipping was added to the model. Whole‐brain differences between the amputees and control group were tested. *Z*‐statistic images were thresholded using clusters determined by *Z* > 2.3, and a familywise error–corrected cluster significance threshold of *p* < 0.05 was applied to the suprathreshold clusters.

We used the post stimulation body localizer scans to assess the neural correlates of stimulation‐induced PLP relief in the intervention condition. We performed a whole‐brain regression analysis, using the offline PLP measure (ie, the difference score used to assess PLP at the end of the experimental session, ∼90 minutes after stimulation offset) as an interparticipant regressor. To further examine S1/M1 missing hand activity, we conducted a region of interest (ROI) analysis (see below). Averaged percentage signal change was extracted from all voxels underlying the missing hand ROI for each amputee in both the sham and intervention conditions.

For presentation purposes, statistical parametric activity maps were projected on the inflated surface using Connectome Workbench (http://www.humanconnectome.org).

#### S1/M1 Hand ROI

For several analyses, individual time series were extracted using a primary sensorimotor missing hand cortex ROI. To define this ROI, a large mask was drawn around the anatomical hand knob, extending the pre‐ and postcentral gyrus and the central sulcus. Within this mask, the top 400 voxels of the controls’ average spatial map for nondominant hand versus foot movements were combined to form the missing hand S1/M1 ROI. Note that our methods did not allow us to reliably dissociate S1 from M1 spatially.

#### Resting State Scans

Similar preprocessing steps were applied as detailed above. Time series, extracted from the missing hand ROI, were used as individual “seeds” to model the activation time series for a further first‐level FEAT analysis. Cerebral spinal fluid (CSF) and white matter (WM) masks were created by performing a structural segmentation using FAST on the MNI standard image. To reduce noise artifacts, CSF and WM scanwise time series were added to the model as nuisance regressors. Head motion parameters were included as further nuisance regressors. Volumes with an estimated absolute mean displacement > 1mm were scrubbed to remove residual motion effects (maximum percentage of volumes scrubbed in a scan = 1%). We then conducted a functional connectivity seed analysis and extracted the averaged percentage signal change from all voxels underlying anatomical left and right insular ROIs (ROIs can be downloaded from https://osf.io/4a5zg/).

#### Stimulation Scans

Similar preprocessing and analysis were applied for the functional scans obtained during stimulation as detailed above, with the following adjustments. To comply with the changed acquisition protocol, spatial smoothing using a 5mm FWHM Gaussian kernel and high‐pass temporal filtering with a cutoff of 150 seconds were applied. Volumes with an estimated absolute mean displacement > 1.5mm (half of the functional voxel size) were scrubbed (maximum percentage of volumes scrubbed in a scan = 2%). CSF and WM scanwise time series were extracted using the masks described in MRI Analysis/Resting State Scans and added to the model as nuisance regressors to reduce noise artifacts.

Group level analysis was carried out similarly to the body localizer scans. The defined contrast was phantom hand (only) movements versus rest. To further assess the neural correlates of stimulation‐induced PLP relief, a whole‐brain regression analysis was carried out for the intervention condition, using the offline PLP measure (ie, the difference score that assessed PLP changes at the end of the experimental session, ∼90 minutes after stimulation offset) as an interparticipant regressor. This allowed us to determine which brain areas, activated during stimulation, predicted subsequent PLP relief. This analysis was done using both flipped (with a regressor of no interest for brain flipping added to the model) and nonflipped brains to investigate brain regions with lateralization to the body (see Fig. 4) and the left/right hemispheres (see Fig. 6).

We next explored which target regions during stimulation predicted the reduction in S1/M1 missing hand activity observed after stimulation. We extracted each individual's activity levels in the S1/M1 missing hand cortex post stimulation (see Fig. 5A). This cluster was used because downregulation of activity in this region after stimulation was predictive of PLP relief. We then used these activity levels as an interparticipant regressor in a whole‐brain group analysis of phantom hand movement activity levels during intervention stimulation.

### *Statistical Analysis*

Statistical analysis was performed using SPSS (v21; IBM, Armonk, NY) and MATLAB (v9.1; MathWorks, Natick, MA). Outliers (>3 between‐subject standard deviations) were replaced with within‐participant means. Using this criterion, one outlier was identified in the PLP ratings and treated as a missing data point accordingly (though note that including this outlier in the analysis did not affect our main outcome). Standard approaches were used for statistical analysis, as mentioned in the Results section. If normality was violated (Shapiro–Wilk *p* < 0.05), nonparametric tests were utilized. To correct for multiple comparisons, α (normally set at 0.05) was divided by the number of comparisons made. For the 1‐sample *t* tests in the PLP analysis (ie, used to assess whether a PLP change was >0), α was adjusted to 0.025. We further report adjusted α when relevant. PLP effect sizes are reported using Cohen *d* or *r* in case of deviations from normality.

### *Role of the Funding Source*

The funders had no role in the study design, data collection, analysis and interpretation, or writing of the report. The corresponding author had full access to all the study data and had final responsibility for the decision to submit for publication.

## Results

### *NIBS Prevents Movement‐Induced PLP Increases*

Immediately after stimulation offset, PLP significantly increased in the sham condition (1‐sample *t*_[13]_ = 4.81, *p* < 0.001, *d* = 1.29), consistent with reports showing that phantom hand movements can increase PLP.^37^ No such PLP increase was observed in the intervention condition (1‐sample Wilcoxon *Z* = −1.73, *p* = 0.084, *r* = −0.46; see Fig 1B). This resulted in a significant difference in PLP modulation between the intervention and sham conditions immediately following stimulation (Wilcoxon *Z* = −2.73, *p* = 0.006, *r* = −0.73; see Supplementary Table 4 for raw pre‐ and post‐PLP means and standard deviations).

### *NIBS Relieves PLP*

At the end of the experimental session (∼90 minutes after stimulation offset; see Fig 1B), PLP remained significantly increased in the sham condition (1‐sample *t*_[13]_ = 2.77, *p* = 0.016, *d* = 0.74). Conversely, we observed significant PLP relief in the intervention condition (1‐sample *t*_[14]_ = −6.20, *p* < 0.001, *d* = −1.60), leading to a significantly different PLP modulation between the intervention and sham conditions (paired *t*_[13]_ = 8.18, *p* < 0.001, *d* = 2.19; see Table 2 for percentage change). Note that our pain effect was specific to PLP and did not generalize to mechanical pain sensitivity, as assessed using PinPrick probe testing (Supplementary Table 5).

**Table 2:** Noninvasive Brain Stimulation–Induced PLP Relief Expressed as a Percentage

Treatment	Immediately after tDCS	End of Experimental Session	After > 6 Days
Sham	+42.9^a^	+28.3^b^	+1.2
Intervention	−6.1	−20.4^a^	−29.5^c^
PLP relief estimate (ie, intervention, considering sham)	−49	−48.7	−30.7

Percentage change was calculated using the raw PLP ratings (ie, before regressing out chronic PLP). A percentage change was calculated between the averaged pre‐ and post‐stimulation scores as follows: ([poststimulation PLP − prestimulation PLP] / prestimulation PLP) * 100. The table shows the PLP modulation in percentages for the sham and intervention conditions separately, as well as a further PLP relief effect size estimate (the intervention stimulation effect controlled for by the sham stimulation effect). We recognize that the intervention condition could both relieve PLP (with respect to baseline) and prevent PLP (with respect to the sham condition). Therefore, this PLP relief effect size estimate combines the intervention effect with the sham effect: That is, we added the effect size of the sham condition to the effect size of the intervention condition. Footnotes indicate significant PLP relief, as shown in Figure 1 and described in the Results section. Note that no statistical tests were carried out for the PLP relief estimate, and as such, no further notation of statistical significance is presented here.

^a^Corrected *p* < 0.001; ^b^corrected *p* < 0.05; ^c^corrected *p* < 0.01.

PLP = phantom limb pain; tDCS = transcranial direct current stimulation.

### *NIBS‐Induced PLP Relief Lasts for at Least 1 Week*

To assess longer‐term PLP effects of intervention stimulation, 2 approaches were taken. First, PLP ratings were assessed in the following session, taken at least 1 week after each stimulation condition, with respect to baseline ratings (as above). Whereas no PLP change was observed for the sham condition (1‐sample Wilcoxon *Z* = 0.00, *p* = 1.00, n = 11, *r* = 0), PLP remained significantly reduced following intervention stimulation (1‐sample Wilcoxon *Z* = −2.80, *p* = 0.005, *r* = −0.89, n = 10; see Fig 1B). Statistical analysis, however, showed no significant difference between the intervention and sham conditions (Wilcoxon *Z* = −1.36, *p* = 0.173, *d* = −0.56). This may be due to the reduced number of participants (n = 6) that could be included in this across‐stimulations analysis, as a result of the counterbalancing of session order.

We further assessed longer‐term intervention effects using daily PLP ratings obtained throughout the week following each stimulation condition, allowing us to study all participants. In accordance with the above, average PLP was significantly lower in the week after intervention stimulation compared to sham (paired *t*_[14]_ = 2.65, *p* = 0.019, *d* = 0.68; see Fig 1C, D).

### *Phantom Hand Movement Activity and Functional Connectivity at Baseline*

**Figure 2:**
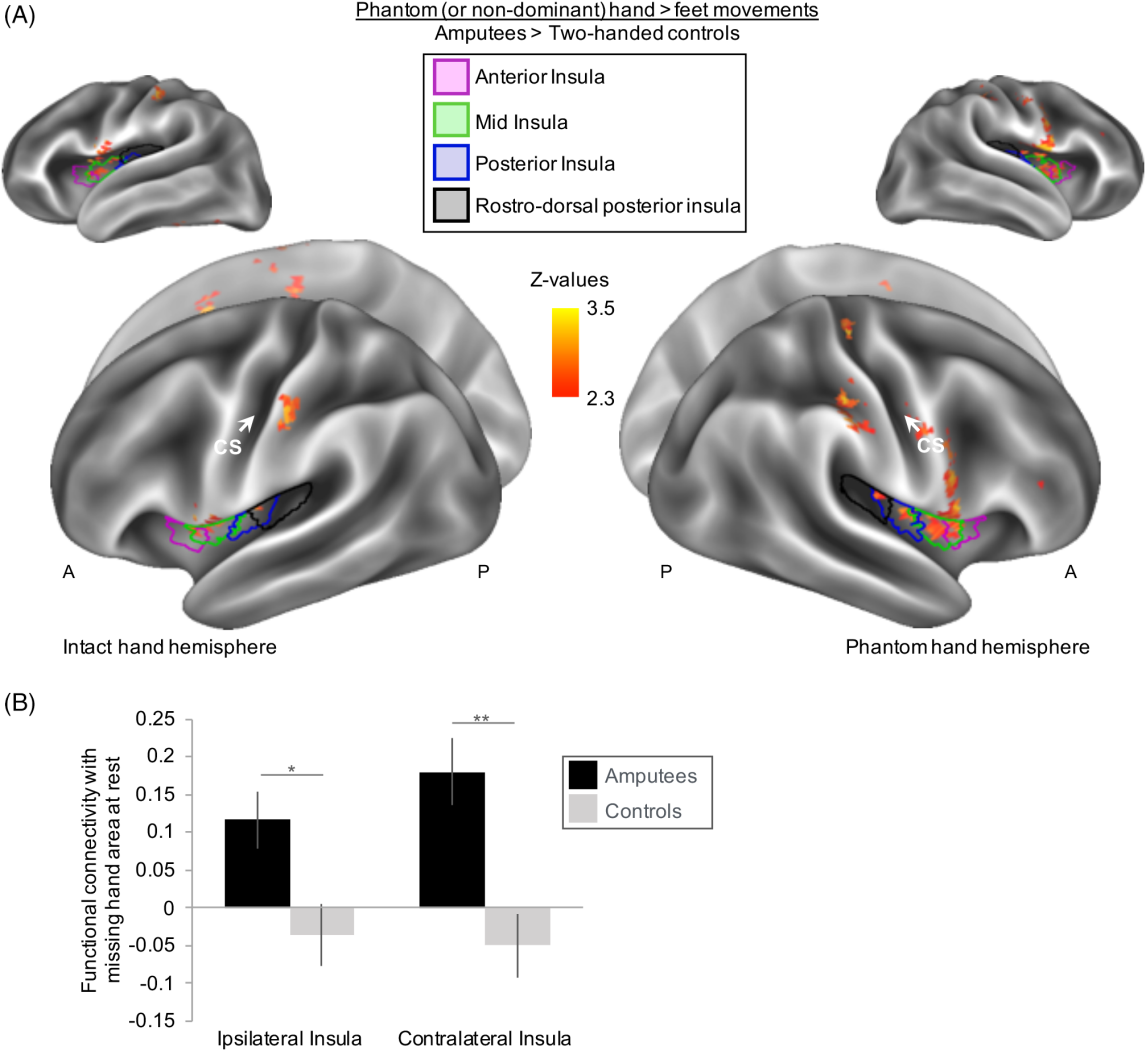
Hyperactivity and hyperconnectivity of amputees’ insular cortex at baseline. (A) Task‐related activity during phantom hand (or nondominant hand for controls) movements were compared between amputees and controls. Results are projected onto an inflated brain template. Amputees showed an increased blood oxygenation level–dependent (BOLD) response (hyperexcitability) in bilateral insula during phantom hand movements compared to 2‐handed controls moving their nondominant hand. (B) Functional connectivity at rest was examined by means of a “seed” of the primary sensorimotor missing hand cortex and compared between amputees and controls. A region of interest (ROI) analysis was implemented to examine resting state functional connectivity between the primary sensorimotor missing hand cortex and the insula ipsilateral and contralateral to the missing hand. Averaged percentage signal change was extracted from all voxels underlying anatomical left and right insular ROIs. Amputees showed increased functional connectivity at rest (hyperconnectivity) between the primary sensorimotor missing hand cortex and bilateral insula, compared to 2‐handed controls. Intact hand hemisphere refers to the hemisphere contralateral to the intact hand. Phantom hand hemisphere refers to the hemisphere contralateral to the missing hand. Ipsilateral/contralateral is with respect to the missing hand side. A = anterior; CS = central sulcus; P = posterior; * = corrected *p* < 0.05; ** = corrected *p* < 0.01. [Color figure can be viewed at www.annalsofneurology.org]

First, we inspected phantom hand movement activity at baseline. Phantom hand movements activated the S1/M1 missing hand cortex, and this maintained phantom hand activity positively associated with chronic PLP (*r*_[13]_ = 0.55, *p* = 0.04), as has previously been shown.^27^, ^29^, ^37^, ^38^, ^40^, ^42^ No phantom hand movement activity differences were found between amputees and controls in the S1/M1 missing hand cortex, but amputees showed increased activity (hereafter hyperactivity) in the bilateral mid, posterior, and rostrodorsal posterior insula (Fig 2), as shown before.^27^, ^29^ Baseline functional connectivity was increased between the S1/M1 missing hand area and bilateral insula in amputees, compared to controls (see Fig 2; hereafter, hyperconnectivity; *t*_[28]_ = −2.75, *p* = 0.01 and *t*_[28]_ = −3.82, *p* = 0.001, corrected α = 0.025, between the S1/M1 missing hand cortex and insula ipsilateral/contralateral to the phantom hand, respectively).

### *Neural Correlates of PLP Relief*

We next analyzed neural activity elicited by phantom hand movements, performed during and after intervention stimulation. All neuroimaging analyses aimed at identifying the neural correlates of PLP relief focused on PLP measures obtained at the earliest time point at which significant PLP relief was observed (ie, the offline ratings; ∼90 minutes after stimulation offset; see Fig 1A).

### *Maintained Phantom Hand Representation after Stimulation*

**Figure 3:**
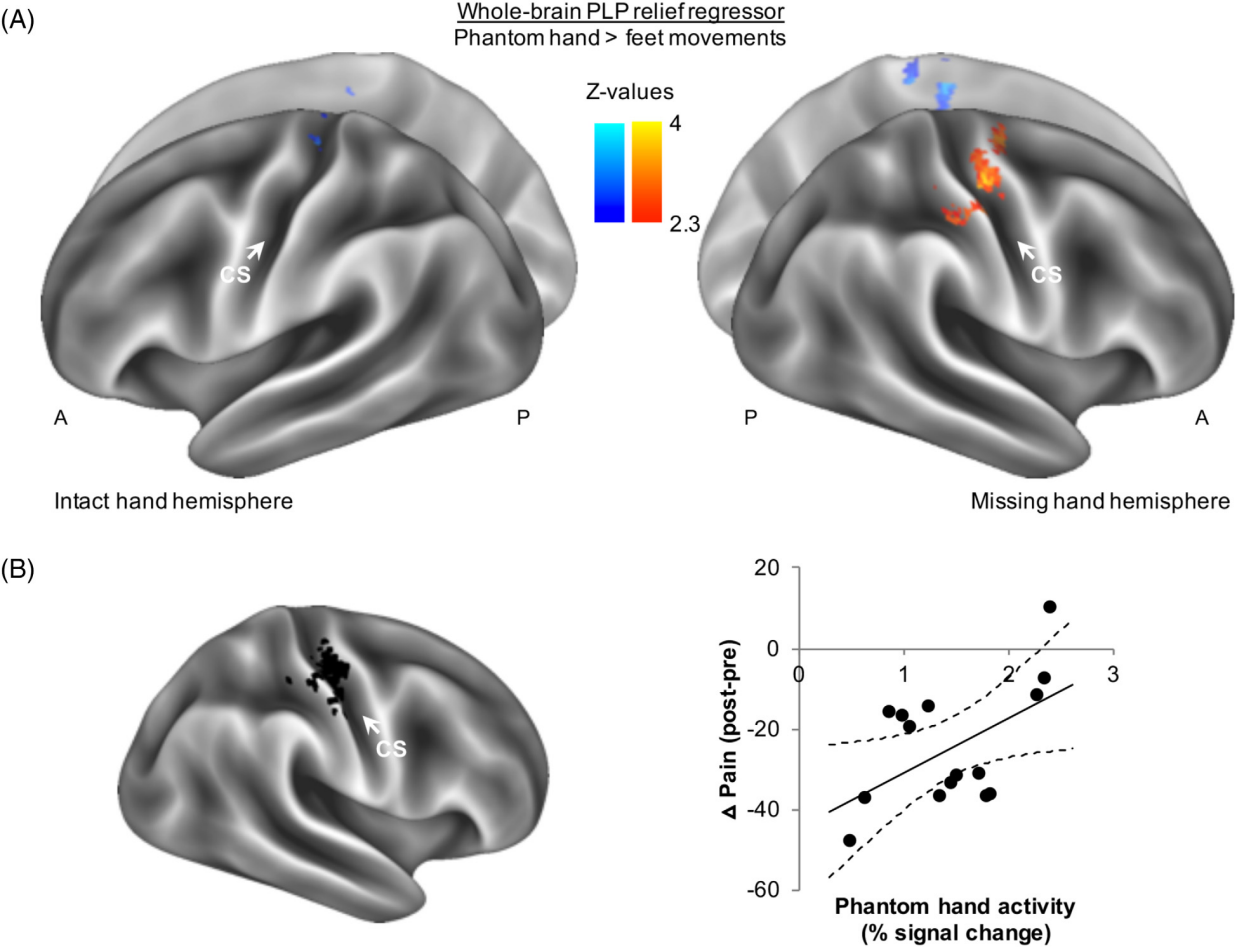
Downregulation of phantom hand activity reflects phantom limb pain (PLP) relief. (A) To assess whether the neural readout of phantom hand movements after intervention stimulation reflected PLP relief, we carried out a whole‐brain regression analysis. Phantom hand movement activity levels after intervention stimulation were modeled using PLP relief scores taken at the end of the experimental session (∼90 minutes after stimulation offset) as a regressor. Results are projected onto an inflated brain template. Voxels showing a positive relationship with PLP relief scores are indicated using the shades in the right colorbar (hot), and voxels exhibiting a negative correlation are indicated using the shades in the left colorbar (cold). Lower activity levels around the primary sensorimotor missing hand cortex correlated with greater PLP relief. (B) An independent region of interest analysis of the primary sensorimotor missing hand cortex (shown in black on an inflated brain model) confirmed that amputees with greater PLP relief (negative values on y‐axis) showed less phantom hand movement activity in the missing hand cortex after intervention stimulation (measured as percentage signal change; x‐axis). The scatter diagram is fitted with a regression line and associated 95% confidence intervals. A = anterior; CS = central sulcus; P = posterior. [Color figure can be viewed at www.annalsofneurology.org]

We first conducted a whole‐brain group regression analysis for PLP relief. We found that following stimulation, amputees who experienced greater PLP relief showed less activity (hereafter “downregulation”) in S1/M1 contralateral to the missing hand (Fig 3A).

This was further confirmed using an ROI of the S1/M1 missing hand cortex. Amputees experiencing greater PLP relief after intervention stimulation showed more downregulation of S1/M1 missing hand cortex activity after stimulation (*r*_[13]_ = 0.54, *p* = 0.036; see Fig 3B). This is consistent with our previous and current baseline results, showing that amputees who have stronger maintained activity in this region during phantom hand movements have worse chronic PLP.^27^, ^29^ No such relationship was observed in the sham condition (*r*_[12]_ = −0.18, *p* = 0.542), resulting in a significant difference in correlations between phantom hand activity levels and PLP scores between the intervention and sham conditions (Fisher *Z* = 1.98, *p* = 0.047).

### *The Role of Pain‐Related Areas during Stimulation*

**Figure 4:**
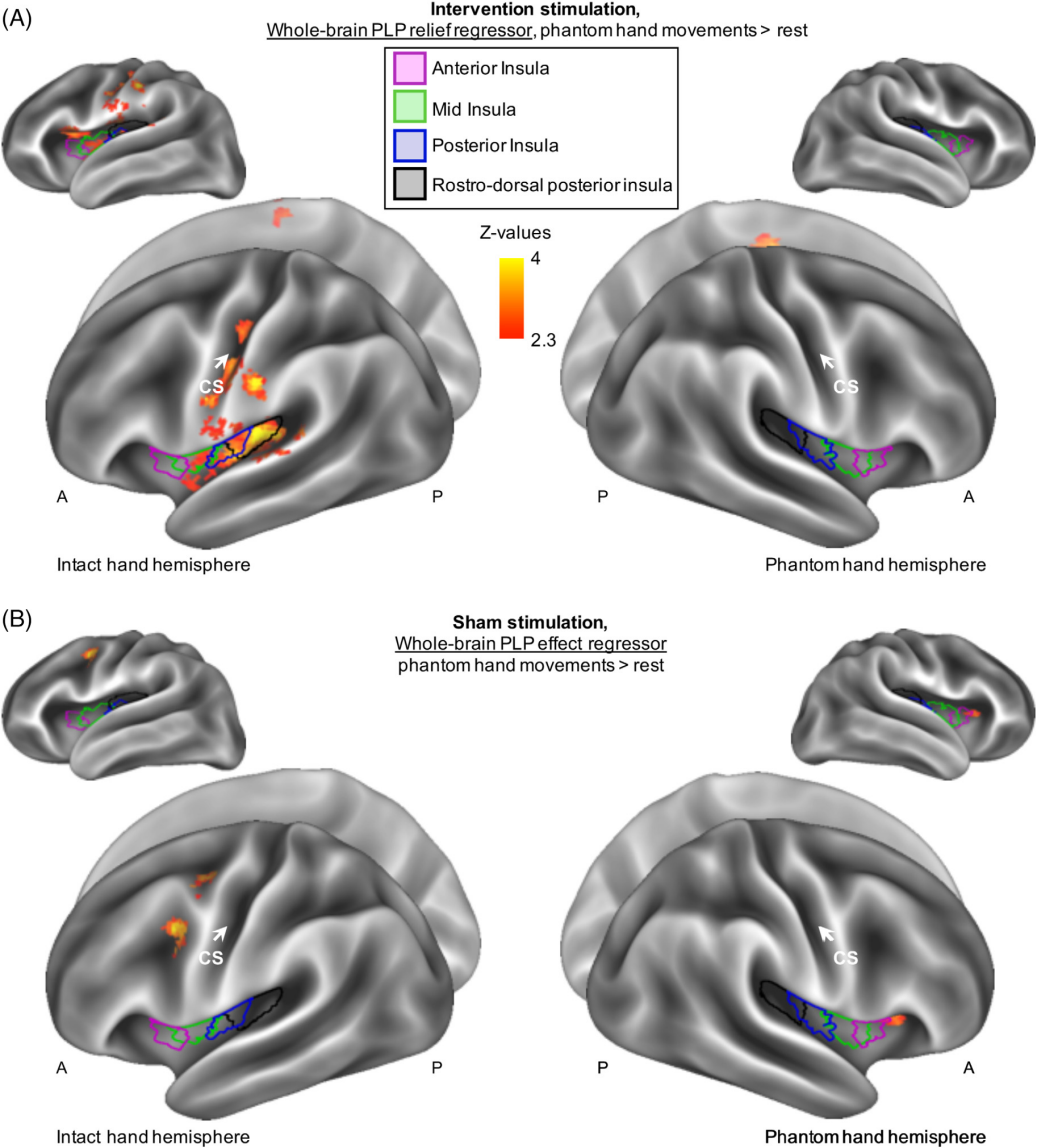
Phantom limb pain (PLP) relief is associated with increased insular recruitment during intervention stimulation. (A) To assess the neural correlates of PLP relief, we carried out a whole‐brain regression analysis of phantom hand movement activity during intervention stimulation, using PLP relief as measured at the end of the experimental session as a regressor. Results are projected onto an inflated surface template. Increased activity in the ipsilateral insula, S2, and other pain‐related areas during stimulation was predictive of subsequent PLP relief. See Supplementary Table 6 for the location of functional peak activations. (B) No significant relationship between PLP and insula activity was observed during sham stimulation, with the exception of a cluster partially overlapping with the anterior aspect of anterior insula. A = anterior; CS = central sulcus; P = posterior. [Color figure can be viewed at www.annalsofneurology.org]

We next explored which brain areas during stimulation were predictive of the PLP relief observed ∼90 minutes after stimulation. Interestingly, we found that increased activity in the ipsilateral (ie, to the missing hand) mid, posterior, and rostrodorsal posterior insula and secondary somatosensory cortex (S2) during intervention stimulation predicted PLP relief (Fig 4A, Supplementary Table 6). No such relationship existed between PLP and insular activity during sham stimulation (see Fig 4B).

**Figure 5:**
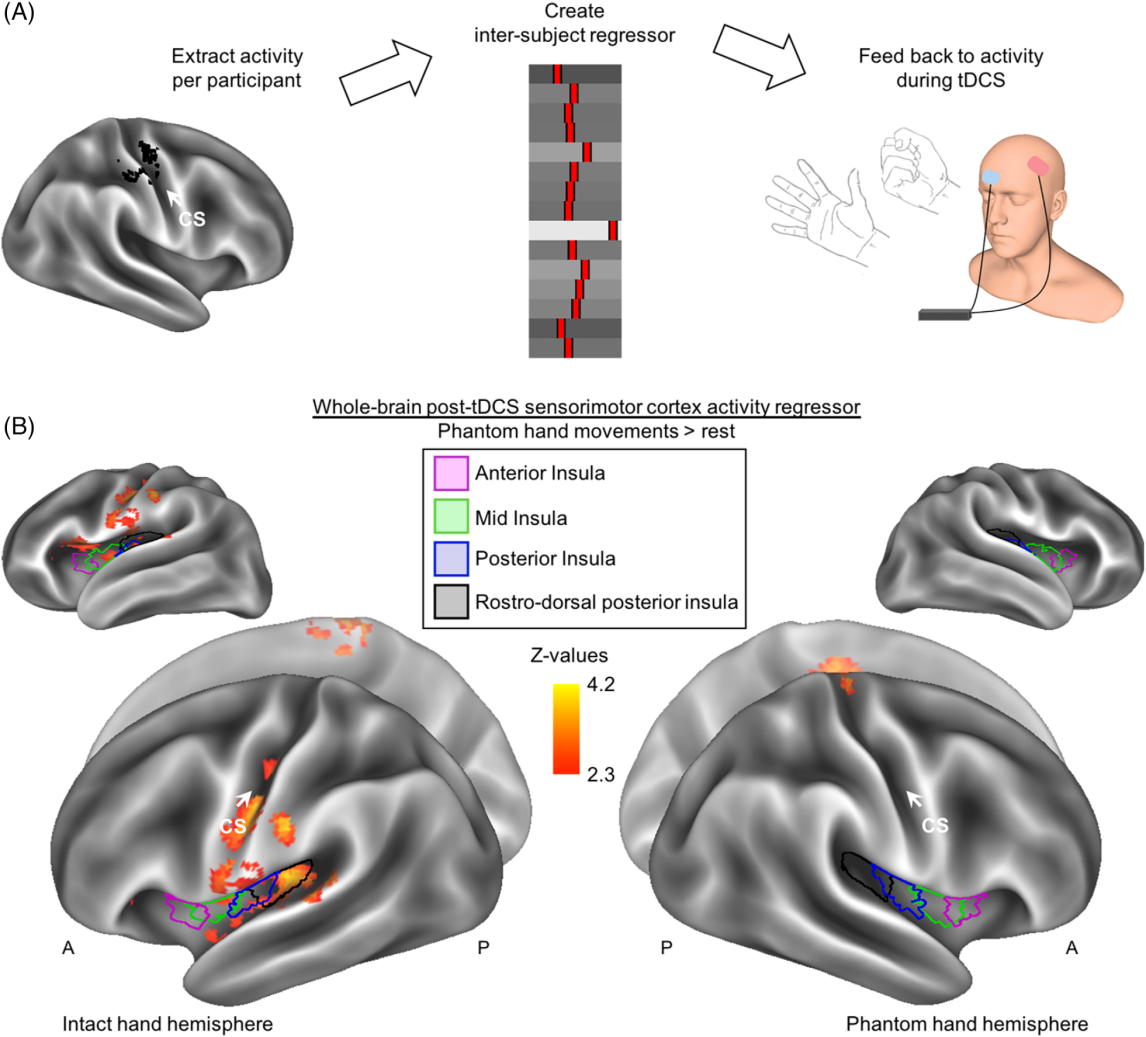
Increased insular activity during stimulation is associated with downregulation of sensorimotor phantom hand activity after stimulation. (A) To assess what neural processes during intervention stimulation predicted the subsequent downregulation of sensorimotor cortex activity, we carried out a whole‐brain regression analysis. To create our regressor, we first extracted the phantom hand movement activity levels post‐stimulation within the cluster shown in Figure 3A and created an interparticipant regressor. This was then used as a whole‐brain regressor of phantom hand movement activity during intervention. (B) Voxels showing a negative correlation are shown (no positive correlation was found). Increased activity in the ipsilateral (ie, to the missing hand) mid, posterior, and rostrodorsal posterior insula, S2, and S1, as well as the bilateral posterior cingulate gyrus, cingulate gyrus, and supplementary motor cortex during task‐concurrent intervention stimulation was predictive of a subsequent downregulation of activity in the primary sensorimotor missing hand cortex after stimulation (see Figs 4 and 6 for related results). A = anterior; CS = central sulcus; P = posterior; tDCS = transcranial direct current stimulation. [Color figure can be viewed at www.annalsofneurology.org]

We then explored which target regions during stimulation predicted subsequent (ie, after stimulation) downregulation of S1/M1 missing hand cortex activity, using a whole‐brain group regression analysis (Fig 5A). We found peaks in the ipsilateral mid‐, posterior, and rostrodorsal posterior insula, S2, S1, bilateral posterior cingulate gyrus, cingulate gyrus, and supplementary motor cortex (see Fig 5B). The magnitude of activity increase during intervention stimulation in these regions predicted the magnitude of activity decrease in the S1/M1 missing hand area after intervention stimulation.

**Figure 6:**
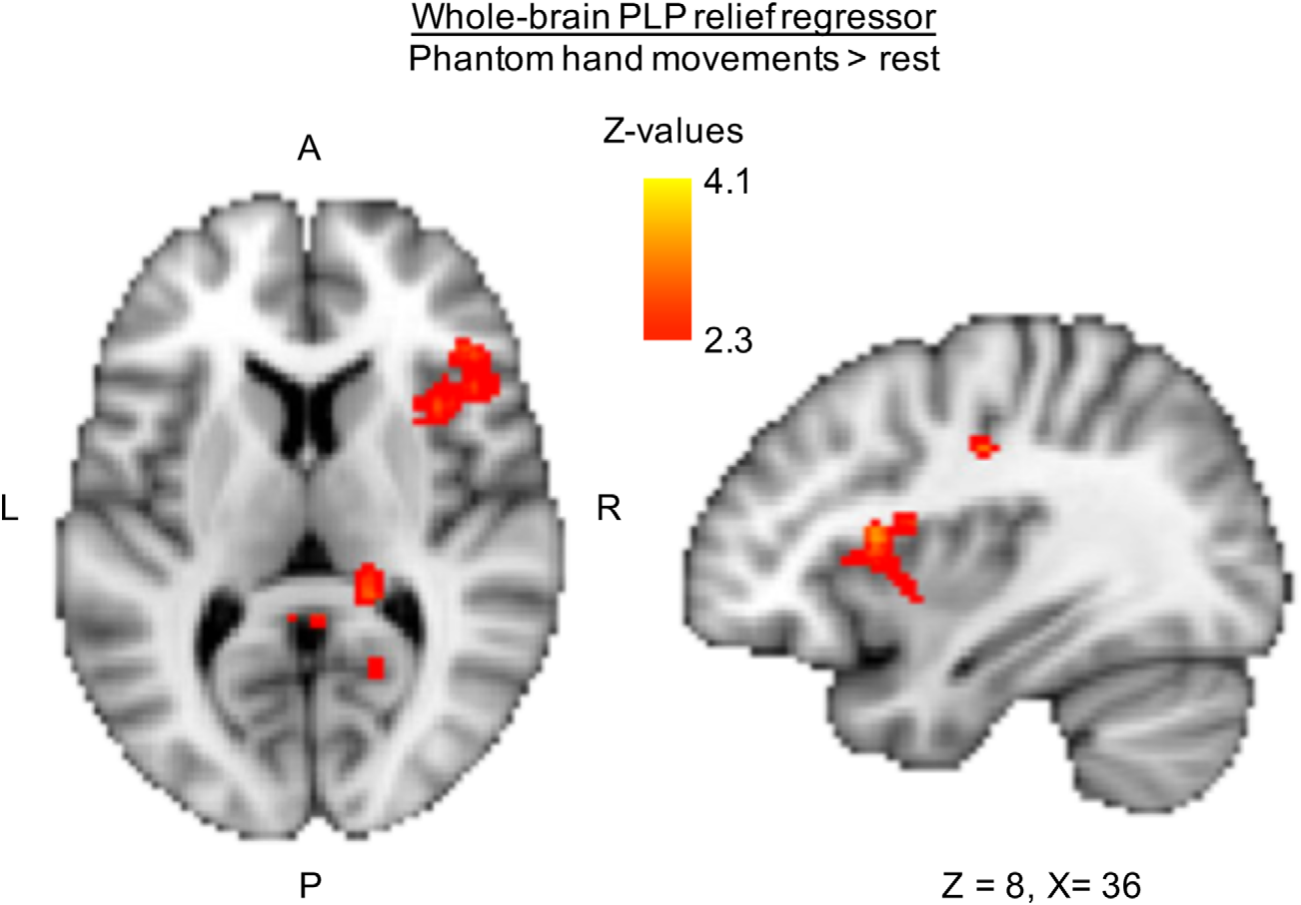
Increased right insular recruitment during stimulation predicts subsequent phantom limb pain (PLP) relief. To assess the neural correlates of PLP relief in brain regions that adhere to right/left lateralization, as opposed to ipsilateral/contralateral lateralization, we repeated the analysis described for Figure 4A in brains that were not flipped (ie, the primary sensorimotor missing hand cortex was not aligned). In short, we conducted a regression analysis using PLP relief scores taken at the end of the experimental session (∼90 minutes after stimulation offset) as a whole‐brain regressor of phantom hand movement activity during intervention stimulation. Increased right insular recruitment during task‐concurrent intervention stimulation was predictive of subsequent PLP relief. A = anterior; L = left; P = posterior; R = right; tDCS = transcranial direct current stimulation. [Color figure can be viewed at www.annalsofneurology.org]

We further repeated the original analysis on the nonflipped brains to investigate regions with lateralization to the left/right hemispheres. This showed that the correlation between insular activity during intervention stimulation and PLP relief was confined to the right hemisphere (Fig 6). Together, our results indicate the involvement of the mid, posterior, and rostrodorsal posterior insula and S2 in inducing PLP relief.

## Discussion

Here we report that a single 20‐minute session of task‐concurrent NIBS (anodal tDCS) over the S1/M1 missing hand cortex caused both short‐ and longer‐term PLP relief. Intervention stimulation prevented movement‐induced PLP immediately after stimulation and subsequently relieved PLP, with effects lasting for at least 1 week. The magnitute of the analgesic effect was 30 to 50%, consistent with other widely used (eg, pharmacological) neuropathic pain relief therapies, suggesting that this effect is clinically relevant (per standards set by the International Association for the Study of Pain).^1^

In addition, we reveal the neural correlates of stimulation‐induced PLP relief. PLP relief correlated with reduced S1/M1 phantom hand activity after intervention stimulation. Both PLP relief and reduced S1/M1 activity were predicted by increased activity in pain‐related brain areas during task‐concurrent intervention stimulation (ie, mid, posterior, and rostrodorsal posterior insula, S2, and S1 ipsilateral to the missing hand, as well as the bilateral posterior cingulate gyrus, cingulate gyrus, and supplementary motor cortex). By providing insight into the mechanisms underlying NIBS‐induced PLP relief, we broaden the mechanistic understanding of PLP and open up new avenues for designing targeted therapies for neuropathic pain, including PLP.

Whereas single tDCS sessions have previously been shown to alleviate neuropathic pain in the short term (ie, < 90 minutes), lasting benefits over days have commonly been achieved only after multiple sessions.^5^, ^12^, ^43^ Although not explicitly tested, our longer‐term intervention efficacy might be due to the use of a task‐concurrent stimulation protocol, designed to activate pathways associated with the readout of aberrant peripheral inputs. This approach may have allowed better targeting of task‐relevant brain regions and thus attained longer lasting PLP relief. However, we cannot determine whether PLP relief was achieved by affecting neural processing of peripheral phantom hand movement signals, or by restituting the normal cortical functioning in a top‐down manner.

PLP relief significantly correlated with reduced activity in the S1/M1 missing hand cortex after intervention stimulation. This is highly compatible with our previous findings, linking greater S1/M1 phantom hand activity with worse chronic PLP.^27^, ^29^ A key aspect of our paradigm was the use of active phantom hand movements to probe the missing hand representation. Because phantom hand movements have been linked with both PLP and PLP relief,^6^, ^37^, ^44^ we verified that the analgesia observed in the intervention condition did not result from differences in task performance during stimulation.

Reduced activity in the S1/M1 missing hand area was only observed after stimulation, suggesting it may be a correlate of PLP relief rather than its driver. We therefore explored which brain regions during stimulation predict PLP relief and this PLP‐related S1/M1 brain activity. Activity changes during intervention stimulation in several pain‐related regions, including the insula, predicted both PLP relief and postintervention downregulation of phantom hand activity in S1/M1. Amputees recruiting the ipsilateral mid, posterior, and rostrodorsal posterior insula more during stimulation showed both greater PLP relief and greater reduction in S1/M1 missing hand activity after stimulation. A wealth of past and recent convergent evidence indicates a key role for the insula (particularly the rostrodorsal posterior portion) in encoding the sensory aspects of pain perception and its modulation.^45^, ^46^, ^47^ However, the role of the insula in PLP has remained largely unexplored.

One might argue that as a core region in pain perception, the reported involvement of the insula (and other core pain‐related areas) could simply reflect ongoing PLP, rather than generation of PLP relief. However, several results in this study suggest otherwise. First, increased insula activity during stimulation correlated with PLP relief, rather than a PLP increase. Second, insula activity did not increase during sham stimulation, where PLP increased. Third, insula activity predicting PLP relief and subsequent downregulation of missing hand activity was measured during intervention stimulation, before analgesia was significantly established. Taken together, these findings suggest a more causal role of the insula (and other pain‐related areas) in alleviating PLP, potentially through S1/M1 modulation.

In line with previous tDCS chronic pain studies, we positioned the excitatory electrode over the contralateral S1/M1 (missing hand) cortex (see introductory paragraphs). This may seem to conflict with our prior findings showing that increased S1/M1 missing hand cortex activity relates to chronic PLP experience.^27^, ^29^, ^31^ However, the spatial effects of tDCS expand well beyond the cortex directly underlying the electrodes:^15^, ^17^, ^48^ Beyond a direct excitatory effect on the underlying cortex, anodal tDCS may lead to the restoration of intracortical inhibitory processes^43^, ^49^ or have indirect effects on pain‐modulating structures (eg, the thalamus).^15^, ^50^ Accordingly, we found that anodal stimulation resulted in modulations both within and beyond the stimulation site. The hyperconnectivity between the S1/M1 missing hand area and bilateral insula observed here may have played a key role in the success of intervention stimulation (ie, as a segue to the regulation of PLP through S1/M1 stimulation). Further research is required to understand whether alternative montages directly targeting the insula and S2 would provide superior results.

In conclusion, our findings confirm NIBS as a viable tool for targeting PLP mechanisms and symptoms. Through better understanding of NIBS‐induced PLP relief, there is now new potential for better targeting and implementation of NIBS, for example, by using transcranial random noise stimulation and targeting of the pain pathway.

## Author Contributions

S.K., M.M., J.O., H.J.‐B., I.T., and T.R.M. conceptualized and designed the study. S.K., M.M., D.H.‐S., and T.R.M. acquired and analyzed the data. S.K., M.M., J.O., H.J.‐B., I.T., and T.R.M. drafted the manuscript and figures. All authors approved the final report.

## Potential Conflicts of Interest

Nothing to report.

## Supporting information

Supplementary Tables
