# Supplementary material for: Neural basis of induced phantom limb pain relief
Source: Ann Neurol. 2019 Jan 7;85(1):59–73. doi: 10.1002/ana.25371 (PMC6492189; doi:10.1002/ana.25371)
Supplement: Supplementary file 1 — Supplementary Tables [file ANA-85-59-s001.docx]

**Supplementary Tables**

**Table S1: Number of amputees included per statistical assessment.** Due to participant non-responses, counterbalancing of sessions, or other unforeseen reasons, we were unable to obtain data for each participant in each session and for each test. Specifically, for the assessment of the difference scores used to measure PLP effects lasting one week post stimulation (i.e. PLP > 6 days post stimulation offset *minus* PLP ~90min prior to stimulation onset), a reduced number of amputees could be tested. Because of the counterbalancing of sessions, some of the participants did not return for a further stimulation session following the intervention or sham conditions, precluding this analysis. The number of participants that could be tested using a (non-parametric variant of the) one-samples t-tests for each condition are as follows: intervention condition: n = 10, sham condition: n = 11, control site condition: n = 11, and cathodal condition: n = 10. Furthermore, as mentioned in the main text, longer-term effects of stimulation were also monitored in the week following the stimulation using daily text messages. PLP ratings obtained in the week before the start of the relevant experimental session were used as a baseline for the construction of the timeline depicted in Fig. 1C. If a participant did not respond to the daily text messages for more than 3 days in the week after an experimental session, this participant’s daily PLP ratings data for the condition tested in that experimental session was discarded (number of participants discarded per condition: intervention=0, sham=0, control site=2, cathodal=3).

**Table S2: Pain ratings results for the control site and cathodal stimulation conditions.** To correct for multiple comparisons, an adjusted α of 0.025 was used to determine significance for comparisons of the PLP change in each condition to 0 (i.e. one-samples t-tests). A similar α correction was used for comparing PLP changes between conditions. As in the sham condition, PLP was significantly increased in the control site condition immediately after tDCS offset. No such change in PLP could be observed in the cathodal stimulation condition. This PLP change in the control site condition was significantly different to that observed in the intervention condition. No such difference was observed between the intervention and cathodal stimulation condition. At the end of the experimental session, we no longer observed a change in PLP for the control site or the cathodal stimulation condition. There was a trend towards a significant difference between the intervention and control site condition. The observed PLP changes were significantly different between the intervention and the cathodal stimulation condition. To assess longer-term effects PLP ratings were assessed in the week after each stimulation condition with respect to baseline ratings. No significant PLP change was observed in the control site condition, while PLP was significantly increased in the cathodal stimulation condition. No significant difference in PLP changes was observed between the intervention and the control site condition. A trend towards a significant difference in PLP changes was observed between the intervention and cathodal stimulation condition. Further daily pain ratings data obtained in the week after tDCS demonstrated that PLP was significantly lower in the week after intervention stimulation compared to the control site and cathodal stimulation conditions.

|  | | **Control site** | **Cathodal** |
| --- | --- | --- | --- |
| **Immediately after NIBS offset** | ***One-sample test*** | Increase (t_(13)_ = 3.60, p = 0.003) | No change (t_(14)_ = 060, p = 0.555) |
|  | ***Compared to intervention stim.*** | Different (Z = -2.83, p = 0.005) | Not different (Z = -.85, p = 0.397) |
| **End of experimental session** | ***One-sample test*** | No change (Z = -0.87, p = 0.382) | No change (t_(13)_ = -0.30, p = 0.766) |
|  | ***Compared to intervention stim.*** | Not different (t_(12)_ = 2.06, p = 0.039) | Different (t_(13)_ = -4.25, p = 0.001) |
| **Week after NIBS** | ***One-sample test*** | No change (Z = -1.42, p = 0.155) | Increase (Z = -2.70, p = 0.007) |
|  | ***Compared to intervention stim.*** | Not different (Z = -1.51, p = 0.128) | Not different (Z = -2.20, p = 0.028) |
| **Week after NIBS (daily ratings)** | ***Compared to intervention stim.*** | Different (t_(11)_ = 3.77, p = 0.003) | Different (t_(11)_ = 2.68, p = 0.022) |

**Table S3: Non-invasive brain stimulation induced PLP relief in percentages (effect sizes).** Effect sizes were calculated using the raw PLP ratings (i.e. before regressing out chronic PLP) for the control site and cathodal stimulation conditions. Similar to the effect sizes described in Table 2, the percentage change was calculated between the averaged pre- and post-stimulation scores as follows: *(post stimulation PLP - pre stimulation PLP) / pre stimulation PLP * 100.*

|  | Immediately after tDCS | End of experimental session | After > 6 days |
| --- | --- | --- | --- |
| Control site | +48.7 | -7.4 | +8.0 |
| Cathodal | -1.4 | -10.8 | +43.1 |

**Table S4: Raw pre- and post PLP ratings per stimulation condition.** Exp. = experimental. Note that statistical analysis was performed on the difference (i.e. post-pre) of the PLP scores and after regressing out the influence of chronic PLP.

|  |  |  | **Intervention** | **Sham** | **Control site** | **Cathodal** |
| --- | --- | --- | --- | --- | --- | --- |
| **Immediately after tDCS** | Pre | mean | 25.50 | 17.87 | 19.64 | 24.13 |
|  |  | SD | 27.05 | 24.65 | 24.06 | 28.48 |
|  | Post | mean | 23.93 | 25.53 | 29.21 | 23.80 |
|  |  | SD | 23.72 | 24.55 | 27.63 | 30.53 |
| **At the end of the exp. session** | Pre | mean | 30.80 | 19.27 | 26.07 | 25.57 |
|  |  | SD | 24.73 | 25.85 | 29.23 | 29.47 |
|  | Post | mean | 24.53 | 24.71 | 24.15 | 22.80 |
|  |  | SD | 27.67 | 23.93 | 31.31 | 26.32 |
| **In week post tDCS (daily pain ratings)** | | mean | 26.50 | 30.99 | 33.03 | 33.11 |
|  |  | SD | 21.25 | 22.11 | 20.78 | 25.89 |
| **After > 6 days** | Pre | mean | 30.20 | 18.18 | 25.91 | 30.80 |
|  |  | SD | 26.82 | 18.34 | 31.69 | 32.76 |
|  | Post | mean | 17.30 | 25.00 | 26.09 | 32.64 |
|  |  | SD | 30.51 | 25.59 | 29.12 | 28.12 |

**Table S5: Pain relief effects did not transfer to mechanic pain sensitivity: Quantitative punctate testing results.** To assess mechanical pain sensitivity before and ~70min after stimulation offset, we used quantitative punctate testing. Participants were blindfolded and asked to verbally rate the ‘prickliness’ (scale 0 - 100, ranging from not felt to most prickly sensation imaginable) of PinPrick stimuli applied to the upper arm of the residual limb or to an analogous location of the intact arm. Pinprick stimuli consist of cylinders, containing a pin-like probe, that is pressed onto the participants’ skin in a 90° angle. These stimuli are routinely used for qualitative sensory testing (QST), a technique used to determine sensitivity to touch and pain. We used PinPrick probes (MRC systems) with various weights: 8mN, 16mN, 32mN, 64mN, 128mN, 256mN and 512mN. Participants were asked to lie on their side and expose the target area of the upper arm for Pinprick stimulation. Pinprick stimuli were presented over 5 blocks (one trial per weight probe, presented in a randomised order). The same procedure was repeated twice, first to the intact upper arm and then to the residual upper arm. To analyse the data, we averaged participants ratings across blocks and PinPrick probe weights, resulting in a single value for each participant at each time point and each stimulation condition. Baseline PinPrick pain levels were then subtracted from post-stimulation PinPrick pain ratings for each stimulation condition. Results showed that none of the stimulation conditions induced a significant effect on mechanical pain sensitivity on either the residual or intact upper arm, as assessed using one-sample t-tests or its non-parametric equivalent (see values in Table). Note that to correct for multiple comparisons in the table, the alpha for significance testing was set at 0.0125 (i.e. 0.05/4 stimulation conditions) for both the residual and intact arm testing. We also did not find a significant difference in mechanical pain sensitivity across stimulation conditions (residual upper arm: X^2^_(13)_=0.14, p = 0.99; intact upper arm: X^2^_(13)_=0.78, p = 0.85). This demonstrated that our stimulation effect was specific to PLP and did not generalise to mechanical pain sensitivity.

|  | **Residual arm** | **Intact arm** |
| --- | --- | --- |
| **Intervention** | Z = -1.15, p = 0.25 | Z = -1.49, p = 0.14 |
| **Sham** | Z = -0.94, p = 0.35 | Z = -0.38, p = 0.70 |
| **Control site** | t_(12)_ = -1.33, p = 0.21 | Z = -1.57, p = 0.12 |
| **Cathodal** | t_(12)_ = 0.74, p = 0.47 | Z = -0.63, p = 0.53 |

**Table S6: Functional location peak activations parameters for Fig. 4A.** Ipsilateral hemisphere refers to the hemisphere ipsilateral to the missing hand. Contralateral hemisphere refers to the hemisphere contralateral to the missing hand. X- y- and z coordinates are in MNI space.

|  | **Hemisphere** | **Z-value** | **MNI coordinates**  **x y z** | | |
| --- | --- | --- | --- | --- | --- |
| **Mid insula** | Ipsilataral | 3.6 | -34 | -12 | 8 |
| **Posterior insula** | Ipsilateral | 3.8 | -36 | -22 | 12 |
| **Rostro-dorsal posterior insula** | Ipsilateral | 4.5 | -42 | -24 | 6 |
| **Primary &secondary somatosensory cortex** | Ipsilateral | 4 | -62 | -22 | 34 |
| **Posterior cingulate gyrus** | Bilateral | 3.8 | -2 | -46 | 12 |
| **Cingulate gyrus** | Bilateral | 3.8 | 8 | -10 | 42 |
| **Supplementary motor cortex** | Bilateral | 2.4 | 8 | -6 | 48 |
